# Impact of an educational tool on young women’s knowledge of cervical cancer screening recommendations

**DOI:** 10.1007/s10552-022-01569-8

**Published:** 2022-03-21

**Authors:** Heike Thiel de Bocanegra, Christine Dehlendorf, Miriam Kuppermann, Sitaram S. Vangala, Anna-Barbara Moscicki

**Affiliations:** 1 Department of Obstetrics and Gynecology, School of Medicine, University of California, Irvine, 333 City Boulevard West, Suite 1400, Orange, CA 92868 USA; 2 Department of Family & Community Medicine, School of Medicine, University of California, San Francisco, San Francisco, USA; 3 Department of Obstetrics, Gynecology & Reproductive Sciences, School of Medicine, University of California, San Francisco, San Francisco, USA; 4 Department of Epidemiology & Biostatistics, School of Medicine, University of California, San Francisco, San Francisco, USA; 5 Department of Medicine Statistics Core, David Geffen School of Medicine, University of California, Los Angeles, Los Angeles, USA; 6 Department of Pediatrics, David Geffen School of Medicine, University of California, Los Angeles, Los Angeles, USA

**Keywords:** Cervical cancer, Cancer prevention, HPV vaccine, Online patient education, Cervical cytology testing

## Abstract

**Purpose:**

Current cervical cancer screening guidelines recommend 3-year screening intervals, in contrast to the previous recommendation of annual screening, to prevent over screening and overtreatment. We evaluated the impact of viewing a tablet-based educational tool prior to seeing a clinician on young women’s knowledge and understanding of cervical cancer screening, HPV vaccination follow-up of abnormal pap smears, and comfort in communicating with their providers.

**Methods:**

This cross-sectional study was part of a cluster-randomized study of fourteen primary care clinics from January 2015 to December 2016. We developed the cervical cancer education tool in English and Spanish using a community-based approach that included formative work and cognitive interviewing. Clinics were randomized to use the intervention (tablet-based patient education tool) or to participate as a control group. We administered surveys to a convenience sample of 229 English- or Spanish-speaking women aged 19 to 35 years in these clinics. We used descriptive analyses and logistic regression models with cluster-robust standard errors to compare differences among the two groups.

**Results:**

Compared to women seen in control clinics, women seen in intervention clinics demonstrated greater knowledge regarding human papilloma virus (HPV (*p* = 0.004) and understanding (*p* < 0.001) of cervical cancer screening. Comfort in communicating with providers was not statistically different (*p* = 0.053). Women in the intervention group felt that the tool helped them understand that an abnormal Pap smear does not require immediate treatment (61.5%).

**Conclusion:**

Innovative online patient education that is offered prior to patients’ interaction with their clinicians can improve their knowledge about cervical cancer prevention and treatment.

**Supplementary Information:**

The online version contains supplementary material available at 10.1007/s10552-022-01569-8.

## Introduction

Cervical cancer screening has resulted in a dramatic decline in the rate of cervical cancer in the USA [1] and is appropriately considered a public health victory. Prior to 2016, annual screening had been recommended. In that year, several national professional agencies reviewed and updated their screening guidelines and recommendations regarding management of abnormal cytology and histology [2–8]. These changes were prompted by improved understanding of the natural history of precancerous changes and cancers, including the high rate of spontaneous regression of lower-grade intra-epithelial lesions and the slow rate of progression to cancer after initial infection [9, 10]. The new guidelines recommended a lengthened screening interval of 3 years when using cytology alone and 5 years when using cytology and HPV testing in women 30 years and older. Additionally, for young women with low-grade abnormalities on cytology, recommendations changed to repeat testing (watchful waiting) instead of colposcopies. These guidelines have the potential to decrease the observed negative outcomes of cervical procedures, such as preterm labor [11], while maintaining the benefits associated with identification of precancerous cervical lesions.

Changes in practices and patient attitudes are often slow to occur [12, 13]. Providers and patients may not be comfortable with lengthier cervical cancer screening intervals and watchful waiting. Women may prefer annual cervical cytology screening regardless of the new recommendations and perceive removing this access as lower quality of care. Clinicians may therefore be less likely to adhere to recommended lengthier screening intervals due to concerns about patients’ perceptions or their own beliefs. The implementation of less-frequent testing intervals and less invasive follow-up of abnormal cervical cytology results is complicated by the fact that some population groups have inadequate screening rates even with the new guidelines (at least one Pap test in the past 3 years) which continues the development of tools that aim to increase cervical cancer testing [14–16].

In the health care system broadly, a range of strategies tools have been used to increase provider adherence to guidelines [17–19]. Tailored health education interventions within community health settings have been shown to have a positive impact on women’s knowledge and informed decision-making in reproductive health issues [20, 21]. Patients’ ability to ask questions, express their concerns, and state their preferences for care provide an enhanced platform for providers to better understand patient needs and to engage patients in prevention and treatment decisions and can also potentially improve screening adherence [22–24].

Strategies for enhanced patient education should incorporate visual aids, interactive features, and audio and provide clarity on what screening entails for greater accessibility and comprehension among women [25–29]. However, most cervical cancer education interventions require consistent staff or equipment resources and are therefore difficult to scale. This study aimed to address the need for a patient engaged cervical cancer and HPV education through the development of an online interactive patient education tool. The tool aimed to address both gaps in screening and potential over-testing or unnecessary procedures, including follow-up of abnormal cervical cytology testing results.

The tool was developed for use in a cluster-randomized trial that aimed to increase adherence to U.S. guidelines for cervical cancer screening and management of abnormal cytology in women under 30 years of age. In this paper, we present results of a cross-sectional analysis using a subsample of women seeking care in the clinics during the trial. Our primary aim was to assess whether the tool improved women’s perceptions of how well they understood the natural history of HPV and cervical cancer tests as well as their self-confidence in communicating with their providers as related to the new cervical cancer screening guidelines. Secondary questions included whether these perceptions were influenced by preferred language (English/Spanish) and country of birth. In addition, we examined characteristics of the women who continued to prefer annual cervical cancer screening after viewing the tool.

## Methods

The sample population consisted of patients who received services from California’s Family Planning, Access, Care, and Treatment (Family PACT) program, which provides free reproductive health services to low-income residents of California who have no other source of family planning coverage. Enrolled Family PACT providers are Medicaid providers, serving predominantly underserved populations, including communities of color [30].

This cross-sectional study was conducted as part of an evaluation of a patient education tool which was administered in a clustered randomized study of Family PACT clinics from January 2015 to December 2016 [31]. Participating clinics served at least 200 female clients under 30 years of age per year since the tool was specifically targeting this age group. Seven of 14 clinics were randomized to incorporate a patient education tool into clinic visits. At sites randomized to the tool, the tool was administered prior to the patient’s visit with the provider. For all women, the survey was administered after their provider visit but prior to their leaving the clinic.

### Development of the patient education tool

We designed the patient education tool using a community-engaged process, in which we collaborated with community stakeholders and experts in the field, including the National Cervical Cancer Coalition, Latinas Contra Cancer, the California Office of Family Planning, and the American Society for Colposcopy and Cervical Pathology. We also conducted focus groups and cognitive interviews with women recruited from our patient stakeholders. We held three focus groups in English and one in Spanish (total *n* = 25). Participants’ experiences varied from having had no previous cervical cancer screening and having had a history of normal or abnormal cytology to being a cervical cancer survivor. Women were asked to identify fears, concerns, and areas of uncertainty around cervical cancer screening and the new recommendations, as well as potential messages that could be used to address these issues.

Themes expressed by patients included fear of missing a cancer diagnosis, concern that the decrease in the recommended frequency of screening being due to a desire to cut cost at expense of patient safety, misconceptions regarding the relationship between cervical cancer screening, and testing for sexually transmitted infections (STIs). Messages identified by the women as important to convey in the educational tool included details on the screening procedure, review of reproductive anatomy, HPV’s relationship with cervical cancer, reasons for the update to screening intervals, information on the HPV vaccine, and clarification about the fact that screening for STIs screening is distinct from cervical cancer screening.

We used these identified themes to develop a paper prototype for an education tool regarding cervical cancer screening and treatment. In developing the tool, we were informed by the Shared Decision-Making model of patient–provider communication, which is increasingly being used as a means to engage in patient-centered communication, especially around reproductive health decisions. In particular, we sought to ensure that patients had adequate information about their ability to participate in a shared decision-making process regarding whether Pap tests or colposcopies following an abnormal Pap test was the appropriate management approach for them. Another major feature was to emphasize that the tool could enhance women’s ability to and comfort with communicating with their provider about decisions related to cervical cancer prevention and treatment [32, 33]. Three cognitive testing series were then performed, with iterative improvement of the tool between each series to ensure the tool addressed the informational needs of the target population. These cognitive interviews were conducted with 17 women aged 21 to 29 years. This feedback was used to inform development of an interactive, online tool with multimedia components, and the option for audio, which then underwent a final round of cognitive testing in both English and Spanish among women attending the participating clinics. Based on patient input, the tool presented information to two target audiences: (a) women at a well-woman visit and (b) women with an abnormal cervical cytology result. We adjusted syntax and vocabulary to a sixth-grade literacy level and made revisions for accuracy, cultural competency, and health literacy. The tool was audio enabled to be accessible for the visually impaired [34].

The final web version of the tool was reviewed and approved by patient and provider stakeholders. Further piloting was performed at each of the clinics randomized to the patient education tool. After viewing the tool, patients were asked a series of questions on clarity and messaging. Final changes were then made to the tool.

### Survey measures and administration

We developed a survey that included 15 items to address three of the goals of the tool: (1) increased knowledge of cervical cancer prevention, covering reproductive anatomy, natural history of HPV, and HPV vaccine protection (4 items), (2) increased understanding of cervical cancer screening recommendations (5 items), and (3) increased comfort in communicating with providers about reproductive health topics (6 items). Participants were asked to indicate their agreement with statements related to these issues on a 5-point Likert scale ranging from strongly agree to strongly disagree. Additional questions assessed sociodemographic information and health care use (15 items). Women in the intervention group were also asked about their experience with the educational tool and their desire to continue with annual screening (9 items).

We administered the paper survey during project site visits from February 2016 to June 2016. Timing of site visits to the intervention and control sites were based on clinic availability and scheduling by clinic staff. Staff approached all women at the time of check in to assess eligibility and willingness to participate. Staff were not asked to document number of women who refused to be approached. With patient’s consent, research staff proceeded with project orientation and written consent. All 229 women approached by research staff consented to participate in the study.

In clinics randomized to the intervention arm, the education tool was provided to the woman prior to consultation with their providers. In both arms, surveys were self-administered in a private setting within the clinic after the patient’s consultation with their providers.

The study was approved by the University of California Los Angeles and the University of California San Francisco Institutional Review Boards, California's Committee for the Protection of Human Subjects, and the Data Research Committee of the California Department of Health Care Services.

### Statistical analysis

We summarized quantitative variables on the Likert scales using means and standard deviations, and categorical variables using frequencies and percentages. We grouped responses from Likert scales to create binary scores based on responses by strongly agree and agree versus neither agree nor disagree, disagree, and strongly disagree. We made group comparisons between study arms (intervention versus control), primary language (English versus Spanish), and women who did and did not still want annual Pap smears.

We compiled composite scores on the three themes (knowledge of cervical cancer prevention, understanding of screening guidelines, and comfort with provider communication). The items are listed in Table 3. Variables were compared between groups using logistic regression models with robust standard errors, with clustering at the clinic level. Treatment effect heterogeneity was evaluated by testing the interaction between group and primary language using these models. *p* values less than 0.05 were considered statistically significant. Analyses were performed using SAS® version 9.4 [35].

## Results

Ninety-six women in the intervention arm and 133 in the control arm were surveyed as part of the educational tool evaluation. Women were on average 27 years old and the majority were U.S.-born and spoke English as their primary language. Approximately half had some college education. There were no statistical differences between the intervention and control groups by age, place of birth, primary language, and education level (Table 1).

**Table 1:** Sample characteristics of women in the intervention and control arm (*n* = 229)

	Intervention Arm (*n* = 96)	Control arm (*n* = 133)	*p* value
Mean age (range)	27.1 (20–39)	27.0 (19–35)	0.86
Place of birth (%)			0.41
U.S.-born	65.3%	57.6%	
Non-U.S.--born	34.7%	42.4%	
Race/Ethnicity			0.27
Hispanic/Latina	81.7%	89.2%	
Non-Hispanic/Latina	18.3%	10.9%	
Primary language spoken (%)			0.21
Spanish	32.3%	43.9%	
English	67.7%	56.1%	
Attended college			0.50
Some college or more	58.4%	52.1%	
Other	41.6%	47.9%	

Women in the intervention group had a higher level of confidence in their knowledge about HPV and cervical cancer as well as cervical cancer screening than women in the control group (Table 2). Women in the intervention group also demonstrated a trend toward greater comfort in communicating with their provider compared to the control group (Table 2). Scores on individual items are given in Table 3.

**Table 2:** Differences in composite scores for knowledge, understanding of cervical cancer screening, and communication with providers between women in the intervention and control arm (*n* = 133)

Composite score	Intervention (*n* = 96) Mean (SD)	Control (*n* = 133) Mean (SD)	*p* value
Knowledge (4 items)	4.03 (0.65)	3.63 (0.93)	0.004
Understanding (5 items)	4.46 (0.82)	3.82 (1.00)	< 0.001
Communication (6 items)	4.35 (0.65)	4.15 (0.87)	0.056

**Table 3:** Comparison for individual Items used for composite scores between women in the intervention and control arms (*n* = 229)

	Intervention arm (*n* = 96) *n* (%) Mean (SD)	Control arm (*n* = 133) *n* (%) Mean (SD)	*p* value
Knowledge of cervical cancer prevention			
I know where my cervix is located			
Strongly agree/agree	88 (91.7)	101 (75.9)	
Mean (SD)**	4.44 (0.97)	3.89 (1.27)	**0.008**
HPV is the cause of cervical cancer			
Strongly agree/agree	70(72.9)	81(61.4)	
Mean (SD)**	3.82 (0.92)	3.73 (1.19)	0.58
Most HPV infections will go away and not cause cancer			
Strongly agree/agree	61(64.2)	34 (26.0)	
Mean (SD)**	3.74 (1.10)	3.05 (1.33)	**0.004**
The HPV vaccine can help protect women against cervical cancer			
Strongly agree/agree	78 (83.9)	84 (64.1)	
Mean (SD)**	4.14 (0.87)	3.87 (1.22)	0.05
Understanding of cervical cancer screening			
I understand what cervical cancer screening is			
Strongly agree/agree	90 (93.8)	103 (77.4)	
Mean (SD)**	4.49 (0.86)	3.94 (1.20)	** < 0.001**
I understand how often I should have cervical cancer screening			
Strongly agree/agree	89 (92.7)	92 (69.2)	
Mean (SD)**	4.48 (0.87)	3.74 (1.29)	** < 0.001**
I understand what to expect from my doctors visit for cervical cancer screening			
Strongly agree/agree	86 (89.6)	92 (69.7)	
Mean (SD)**	4.43(0.98)	3.7(1.23)	** < 0.001**
I know when I need my next (or first) Pap smear			
Strongly agree/agree	90 (93.8)	102 (76.7)	
Mean (SD)**	4.53 (0.83)	3.98 (1.12)	** < 0.001**
I understand what human papilloma virus (HPV) is			
Strongly agree/agree	87 (90.6)	91 (94.8)	
Mean (SD)**	4.35 (0.88)	3.72 (1.20)	** < 0.001**
Comfort in communicating with providers about reproductive health topics			
Please indicate how comfortable you feel in asking your provider about Pap smears			
Extremely comfortable/comfortable	92 (95.8)	110 (84.0)	
Mean (SD)**	4.33 (0.72)	4.11 (0.95)	0.84
Please indicate how comfortable you feel in asking your provider about cervical cancer screening			
Extremely comfortable/comfortable	90 (93.8)	108 (81.8)	
Mean (SD)**	4.33 (0.68)	4.07 (0.99)	0.33
Please indicate how comfortable you feel in asking your provider about HPV			
Extremely comfortable/comfortable	89 (92.7)	105 (79.5)	
Mean (SD)**	4.31 (0.69)	4.08 (0.96)	0.47
Please indicate how comfortable you feel in asking your provider about HPV vaccine			
Extremely comfortable/comfortable	88 (91.7)	109 (82.6)	
Mean (SD)**	4.3 (0.73)	4.11 (0.92)	0.94
Please indicate how comfortable you feel in asking your provider about birth control			
Extremely comfortable/comfortable	93 (96.9)	119 (90.2)	
Mean (SD)**	4.47 (0.65)	4.27 (0.87)	0.47
Please indicate how comfortable you feel in asking your provider about menstrual problems			
Extremely comfortable/comfortable	91 (94.8)	117 (88.6)	
Mean (SD)**	4.38 (0.80)	4.24 (0.90)	0.41

Bolded text: *p* < 0.01

**Means are calculated from responses on a Likert scale

Tests for treatment effect heterogeneity by primary language did not show any evidence of such heterogeneity for any of the measures. A sizeable portion of women (30.2%) in the patient education tool group continued to express a preference for annual screening. To investigate this further, we examined factors that may have influenced this preference among women in the intervention group. No demographic variables were statistically significant predictors of having a preference for annual screening. However, there was a trend for non-U.S.-born women to prefer annual screening (Table 4).

**Table 4:** Comparison between women who continue to desire annual Paps versus triannual Paps (*n* = 96)

	Women who desire annual Paps (*n* = 29) *n* (%)	Women who accept 3-year intervals (*n* = 67) *n* (%)	*p* value
Mean age	27.93	26.74	0.42
Place of birth			
Non-U.S.-born	14 (48.28)	19 (28.79)	0.15
Primary language spoken			
Spanish	13 (44.83)	18 (27.27)	0.34
Attended college			
Some college or more	12 (44.44)	41 (63.08)	0.20
If I had an abnormal Pap smear this tool helped me feel comfortable with not treating it right away			
Agree/strongly agree	22 (75.86)	40 (60.61)	0.08
This tool helps me to understand that I may not need to treat an abnormal Pap smear right away			
Agree/strongly agree	16 (55.17)	42 (64.18)	0.29
I understand what human papilloma virus is			
Agree/strongly agree	27 (93.10)	60 (89.55)	0.53
I worry about getting cervical cancer			
Agree/strongly agree	18 (62.07)	40 (59.70)	0.62

Women in the intervention group stated that the information in the tool was clear and helped them feel prepared to talk to their doctor (Table 5). The majority of women said the tool helped them understand or feel comfortable with not treating an abnormal Pap smear right away (62% and 65%, respectively). Eighty-eight percent of women expressed satisfaction with the educational tool and ninety-six percent of women would recommend the tool to their friends. No differences were found for responses by primary language spoken.

**Table 5:** The number (%) of women who received the patient educational tool and agree or strongly agree with the following statements (*n* = 96)

Question	*n* (%)*
The information in the tool was clear	93 (96.8)
The tool helped me feel prepared to talk with my health care provider (doctor or nurse) about cervical cancer screening	88 (91.7)
If I had an abnormal Pap smear this tool helped me feel comfortable with not treating it right away	62 (65.3)
This tool helps me to understand that I may not need to treat an abnormal Pap smear right away	59 (61.5)

*Denominators vary due to missing data

## Discussion

This cross-sectional study showed that a bilingual interactive cervical cancer educational tool offered in clinic settings increased women’s understanding of the natural history of HPV, cervical cancer, and current screening guidelines. There was also evidence that using the tool led to greater comfort in communicating with their provider about screening. The latter is important as women who interacted with the tool might be better able to communicate with providers about recommendations, including any concerns they may have about lengthened screening intervals.

These results are consistent with studies evaluating the impact of kiosks, tele-, or foto novellas, and digital stories that found a positive impact on knowledge. In other preventive health areas, interactive multimedia tools emphasizing empowering women were shown to increase patient satisfaction and preventive health behaviors [36–40].

Patients’ ability to ask questions, express their concerns, and state their preference for care provide an enhanced platform for providers to better understand patient needs and to engage women in prevention and treatment decisions [20]. However, some women may not perceive that messages on cervical screening involve a “decision” which limits their ability to weigh options, risks, and benefits that are appropriate for them [41]. Enabling patients to be active partners in cervical cancer screening and follow-up of abnormal test results is crucial for effective cervical cancer prevention. One strength of our tool was its design to be self-paced with an audio option that can be accessed prior to the interaction with the provider either at the clinic or at home or can be used by health educators in it part or its entirety. These elements provide opportunities to scale the intervention and expand its use in communities with geographic and language barriers.

Another strength of this study was the process we used in developing the tool, grounded in a community-based approach that employed extensive engagement with the English- and Spanish-speaking target population, including formative work, cognitive interviewing, and partnership with community organizations. Through this process, we gained insight into both the areas of concern we needed to address in our tool, as well as ideas for how to approach them.

Another innovative feature of our intervention was the diversity of visual content and visual aid which has been shown to increase comprehension, recall, and maintain user attention to positively influence health outcomes [25, 26, 42, 43]. We used videos as well as real and illustrated pictures to describe the HPV natural history, the appearance of abnormal cells, reproductive anatomy, and screening guidelines. In the development process, showing pictures of female anatomy and of the real cervix was considered as one of the most interesting and important aspects of the intervention tool.

Participants in our study considered the tool easy to use. They also reported that the tool helped them to be better prepared to speak to their clinician about cervical cancer and to understand “watchful waiting” after an abnormal cervical cytology test. The impact of the tool seems to be mainly cognitive, as the degree of *comfort* in communicating with their provider on reproductive health issues did not vary significantly from that of women in the control group.

However, even though knowledge about cervical cancer screening guidelines increased after use of the tool, 30 percent of the women in the intervention group preferred to receive annual cervical cytology screenings. This finding is consistent with a 2015 web-based survey in which 30 percent of U.S. women indicated that they preferred annual Pap testing [44]. We could not identify factors that influenced this preference [45, 46], but we observed a trend toward annual screening preference among non-U.S.-born women. This preference may be grounded in their experience in their country of origin, as many low- and middle-income countries have high cervical cancer cervical cancer mortality because of underscreening.

Limitations to this study include its relatively small sample size. Randomization occurred at clinic level and all women present in the clinic the day of our site visit were asked to participate in the study. However, staff was not asked to record the number of women who declined or could not be approached due to clinic flow constraints. Another limitation in the design was that we surveyed women on the same day that they viewed the intervention. It remains unclear if these messages are retained over longer periods of time. In addition, we did not administer the survey prior to the intervention. However, since the women participating were in a study where the clinics were randomized, there is no reason to believe the baseline knowledge would have been different between the two groups. We were also not able to assess the time of exposure to the tool prior to the clinic interaction. Time of exposure and the overall feasibility of a clinic-based approach needs vary by clinic setting. This tool was piloted in settings with “downtime” while rooming patients in the exam room. However, in other clinic settings, it may be better to encourage women to review the tool prior to the clinic visit or to disseminate the tool directly to the community [47]. Future studies need to compare various approaches to engage women in cancer screening and follow-up. Finally, the tool was developed for English- or Spanish-speaking women who were eligible for publicly-funded family planning services. Future studies also should include women with private insurance and women who speak other languages than English or Spanish.

In the context of changing guidelines [48], there is a continued need for enhanced health education in cervical cancer prevention, particularly among ethnic minority and underinsured populations. Strategies for enhanced patient education should incorporate visual aids, interactive features, and audio and provide clarity on what screening entails for greater accessibility and comprehension among women. Self-administered, online education tools emphasizing patient–provider communication that can be implemented in clinic and community settings offer opportunities for increased adherence to appropriate cervical cancer screening guidelines that can benefit women nationwide.

Dissemination of patient education tools around cervical cancer screening and prevention is needed. Messaging around, with an emphasis on the fact that cervical cancer screening is necessary, but that too frequent screening can result in unnecessary procedures that can jeopardize women’s health. Community-engaged education tools such as the one evaluated in this study are critical for supporting patients to navigate their health care experience with agency and health literacy.

## Supplementary Information

Below is the link to the electronic supplementary material.

Supplementary file1 (PDF 438 kb)

Supplementary file2 (PDF 2052 kb)

## Data Availability

Not applicable.
