# Supplementary material for: Impact of an educational tool on young women’s knowledge of cervical cancer screening recommendations
Source: Cancer Causes Control. 2022 Mar 21;33(6):813–21. doi: 10.1007/s10552-022-01569-8 (PMC9085671; doi:10.1007/s10552-022-01569-8)
Supplement: Supplementary file 1 — Supplementary file1 (PDF 438 kb) [file 10552_2022_1569_MOESM1_ESM.pdf]

Clinic Name: ____________________ ID # ____________ Date: ____/____/_____

*You have been selected to participate in a 5-10 minute survey that asks you questions about and experience with cervical cancer screening. There are no right or wrong answers to these questions. You may skip or refuse to answer any questions on this survey. Your answers will help us to improve our care. We appreciate your honest feedback!*

1. **What is your age?** ________

2. **Which one of the following categories best describes your Race?**

| Please tell me which one or more of the following you would use to describe yourself | Check all that apply |
|---|---|
| White[1] | ○ |
| Black or African American[2] | ○ |
| Asian[3] | ○ |
| American Indian or Alaska Native[4] | ○ |
| Native Hawaiian / Pacific Islander[5] | ○ |
| Other (Specify)[6]: ________________________ | |

3. **Are you Latino or Hispanic?** ☐1 Yes ☐2 No (If no, skip to question 5)

4. **Which of the following categories best describes your ethnicity?**
*Please indicate which one or more of the following you would use to describe yourself:*

| If Yes, | Check all that apply |
|---|---|
| Mexican, Mexican-American, Chicano | ○ |
| Salvadoran[2] | ○ |
| Guatemalan[3] | ○ |
| Dominican[4] | ○ |
| Colombian[5] | ○ |
| Puerto Rican[6] | ○ |
| Cuban[7] | ○ |
| Spanish-American (from Spain)[8] | ○ |
| Other Latino[9] | ○ |

5. **What is your primary language spoken at home?**

☐1 English

☐2 Spanish

☐3 Both *(English & Spanish)*

☐4 Other *(Specify)* __________________

6. **In what country were you born?** ________________________________________

7. **In what country were your parents born?** ______________________________

**8. Please mark the box below that indicates the highest grade/degree or years of completed by:**

| a) You | b) Parent 1 | c) Parent 2 | |
|---|---|---|---|
| ☐1 | ☐1 | ☐1 | Never attended school or only attended kindergarten |
| ☐2 | ☐2 | ☐2 | Grades 1 through 8 (Elementary) |
| ☐3 | ☐3 | ☐3 | Grades 9 through 11 (Some high school) |
| ☐4 | ☐4 | ☐4 | Grade 12 or GED (High school graduate) |
| ☐5 | ☐5 | ☐5 | 1 year to 3 years of college (Some college, associates or technical school) |
| ☐6 | ☐6 | ☐6 | 4 years or more years of College (College graduate) |
| ☐7 | ☐7 | ☐7 | Not Applicable / Don't Know |

**9. Where do you usually go <u>when you are sick</u>?** *(Check all that apply)*

☐1 My usual Doctor
☐2 Local Pharmacy
☐3 Local Clinic/ Health center /Hospital
☐4 Health Fair
☐5 Emergency Room
☐6 Alternative medicine practitioner (e.g., natural herbalist, message therapist, acupuncturist, spiritual advisor, or other healing professional)
☐7 No place
☐8 Some other place *(Specify)*: ____________________

**10. Where do you go when you <u>need advice about your health</u>?** *(Check all that apply)*

☐1 My usual Doctor
☐2 Local Pharmacy
☐3 Local Clinic/ Health Center /Hospital
☐4 Health Fair
☐5 Internet/ Mobile Apps
☐6 Alternative medicine practitioner (e.g., natural herbalist, message therapist, acupuncturist, spiritual advisor, or other healing professional)
☐7 My friends and family
☐8 Some other place *(Specify)*: _______________________

**11. Have you ever had a Pap smear before?** ☐1 Yes ☐2 No

**12. Is the Primary reason for your visit today to get a Pap smear?** ☐1 Yes ☐2 No

***<u>INSTRUCTIONS:</u> The following questions ask about your experience using the iPad tool.***

| | | | | |
|---|---|---|---|---|
| **1. Which module did you go through on the *iPad*?** 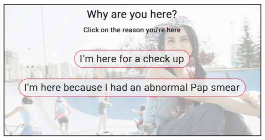 | *English, Regular Check-Up* ☐1 | *English, Abnormal Pap Smear* ☐2 | *Spanish, Regular Check-Up* ☐3 | *Spanish, Abnormal Pap Smear* ☐4 |

| 2. After seeing the tool, how often do you think a woman should get a Pap smear? | Every year ☐ 1 | Every 3 years ☐ 2 | Don't Know ☐ 3 | Not Sure ☐ 4 |
|---|---|---|---|---|

| ***INSTRUCTIONS:*** ***Please indicate to what degree you agree with the following statements. Mark your response with an "X" where appropriate.*** | *Strongly Disagree* 1 | *Disagree* 2 | *Neither Disagree nor Agree* 3 | *Agree* 4 | *Strongly Agree* 5 | *Refuse to Reply* 6 |
|---|---|---|---|---|---|---|
| **3. The information in the tool was clear.** | ☐ | ☐ | ☐ | ☐ | ☐ | ☐ |
| **4. The tool helped me feel prepared to talk with my healthcare provider (doctor or nurse) about cervical cancer screening.** | ☐ | ☐ | ☐ | ☐ | ☐ | ☐ |
| **5. If I had an abnormal Pap smear, this tool helped me feel comfortable with not treating it right away.** | ☐ | ☐ | ☐ | ☐ | ☐ | ☐ |
| **6. This tool helps me to understand that I may not need to treat an abnormal Pap smear right away.** | ☐ | ☐ | ☐ | ☐ | ☐ | ☐ |

| **7. How satisfied were you with the tool?** | Completely Unsatisfied ☐ 1 | Somewhat Unsatisfied ☐ 2 | Somewhat Satisfied ☐ 3 | Very Satisfied ☐ 4 | Completely Satisfied ☐ 5 |
|---|---|---|---|---|---|
| **8. Would you recommend this tool to your friends?** | | | | Yes ☐ 1 | No ☐ 2 |
| **9. Are there any suggestions or comments you have for us about the tool?** (Please write your comments in the space to the right) | | | | | |

| ***INSTRUCTIONS:*** ***Please indicate to what degree you agree with the following statements. Mark your response with an "X" where appropriate.*** | *Strongly Disagree* 1 | *Disagree* 2 | *Neither Disagree nor Agree* | *Agree* 4 | *Strongly Agree* 5 | *Refuse to Reply* 6 |
|---|---|---|---|---|---|---|
| **10. I know where the cervix is located.** | ☐ | ☐ | ☐ | ☐ | ☐ | ☐ |
| **11. I understand what cervical cancer screening is.** | ☐ | ☐ | ☐ | ☐ | ☐ | ☐ |
| **12. I understand how often I should have cervical cancer screening.** | ☐ | ☐ | ☐ | ☐ | ☐ | ☐ |
| **13. I understand what to expect from my doctors visit for cervical cancer screening.** | ☐ | ☐ | ☐ | ☐ | ☐ | ☐ |
| **14. I know when I need my next (or first) Pap smear.** | ☐ | ☐ | ☐ | ☐ | ☐ | ☐ |
| **15. I understand what Human Papilloma Virus (HPV) is.** | ☐ | ☐ | ☐ | ☐ | ☐ | ☐ |

 *Version 2: June 02, 2016*

| ***Section Continued*** | *Strongly Disagree* 1 | *Disagree* 2 | *Neither Disagree nor Agree* 3 | *Agree* 4 | *Strongly Agree* 5 | *Refuse to Reply* 6 |
|---|---|---|---|---|---|---|
| **16. HPV is the cause of cervical cancer.** | ☐ | ☐ | ☐ | ☐ | ☐ | ☐ |
| **17. Most HPV infections will go away and not cause cancer.** | ☐ | ☐ | ☐ | ☐ | ☐ | ☐ |
| **18. The HPV vaccine can help protect women against cervical cancer.** | ☐ | ☐ | ☐ | ☐ | ☐ | ☐ |
| **19. I feel confident that I know what is needed to prevent cervical cancer.** | ☐ | ☐ | ☐ | ☐ | ☐ | ☐ |
| ***The following questions ask about your thoughts about cervical cancer and how they affect you.*** | *Strongly Disagree* 1 | *Disagree* 2 | *Neither Disagree nor Agree* 3 | *Agree* 4 | *Strongly Agree* 5 | *Refuse to Reply* 6 |
| **20. Cervical cancer screening is confusing.** | ☐ | ☐ | ☐ | ☐ | ☐ | ☐ |
| **21. I would worry a lot if my Pap smear was abnormal and it wasn't treated right away.** | ☐ | ☐ | ☐ | ☐ | ☐ | ☐ |
| **22. I worry about getting cervical cancer.** | ☐ | ☐ | ☐ | ☐ | ☐ | ☐ |
| | *Not at all* 1 | *Extremely*2 | *Slightly* 3 | *Moderately* 4 | *Quite a bit*5 | *Refuse to Reply* 6 |
| **23. Overall, during the past month how much have you been bothered by thoughts (or worried) about your chances of getting cervical cancer?** | ☐ | ☐ | ☐ | ☐ | ☐ | ☐ |

| ***24. Please indicate how comfortable you feel <u>talking</u> to your provider about:*** | *Extremely Uncomfortable* | *Uncomfortable*2 | *Neither uncomfortable nor comfortable*3 | *Comfortable* 4 | *Extremely Comfortable* 5 | *Refuse to Reply* 6 |
|---|---|---|---|---|---|---|
| **a. Pap smears** | ☐ | ☐ | ☐ | ☐ | ☐ | ☐ |
| **b. Cervical cancer screening** | ☐ | ☐ | ☐ | ☐ | ☐ | ☐ |
| **c. HPV** | ☐ | ☐ | ☐ | ☐ | ☐ | ☐ |
| **d. HPV Vaccine** | ☐ | ☐ | ☐ | ☐ | ☐ | ☐ |
| **e. Birth Control** **(pills, condoms, IUD, other preventive methods)** | ☐ | ☐ | ☐ | ☐ | ☐ | ☐ |

| ***Section Continued*** | *Extremely Uncomfortable* | *Uncomfortable 2* | *Neither uncomfortable nor comfortable 3* | *Comfortable 4* | *Extremely Comfortable 5* | *Refuse to Reply 6* |
|---|---|---|---|---|---|---|
| **f. Menstrual Problems** | ☐ | ☐ | ☐ | ☐ | ☐ | ☐ |

| ***25. Please indicate how comfortable you feel in <u>asking</u> your provider about:*** | *Extremely Uncomfortable 1* | *Uncomfortable 2* | *Neither uncomfortable nor comfortable 3* | *Comfortable 4* | *Extremely Comfortable 5* | *Refuse to Reply 6* |
|---|---|---|---|---|---|---|
| **a. Pap smears** | ☐ | ☐ | ☐ | ☐ | ☐ | ☐ |
| **b. Cervical cancer screening** | ☐ | ☐ | ☐ | ☐ | ☐ | ☐ |
| **c. HPV** | ☐ | ☐ | ☐ | ☐ | ☐ | ☐ |
| **d. HPV Vaccine** | ☐ | ☐ | ☐ | ☐ | ☐ | ☐ |
| **e. Birth Control (pills, condoms, IUD, other preventive methods)** | ☐ | ☐ | ☐ | ☐ | ☐ | ☐ |
| **f. Menstrual Problems** | ☐ | ☐ | ☐ | ☐ | ☐ | ☐ |

| ***26. I felt I understood what my provider told me about:*** | *Strongly Disagree 1* | *Disagree 2* | *Neither Disagree nor Agree 3* | *Agree 4* | *Strongly Agree 5* | *Not Applicable 6* |
|---|---|---|---|---|---|---|
| **a. Pap smears** | ☐ | ☐ | ☐ | ☐ | ☐ | ☐ |
| **b. Cervical cancer screening** | ☐ | ☐ | ☐ | ☐ | ☐ | ☐ |
| **c. HPV** | ☐ | ☐ | ☐ | ☐ | ☐ | ☐ |
| **d. HPV Vaccine** | ☐ | ☐ | ☐ | ☐ | ☐ | ☐ |
| **e. Birth Control (pills, condoms, IUD, other preventive methods)** | ☐ | ☐ | ☐ | ☐ | ☐ | ☐ |
| **f. Menstrual Problems** | ☐ | ☐ | ☐ | ☐ | ☐ | ☐ |

*YOU'VE REACHED THE END OF THE QUESTIONNAIRE. WE GREATLY APPRECIATE YOUR TIME AND INPUT. PLEASE RETURN THE QUESTIONNAIRE TO THE RESEARCH STAFF. THANK YOU!*



*Version 2: June 02, 2016*
