# Supplementary material for: Impact of an educational tool on young women’s knowledge of cervical cancer screening recommendations
Source: Cancer Causes Control. 2022 Mar 21;33(6):813–21. doi: 10.1007/s10552-022-01569-8 (PMC9085671; doi:10.1007/s10552-022-01569-8)
Supplement: Supplementary file 2 — Supplementary file2 (PDF 2052 kb) [file 10552_2022_1569_MOESM2_ESM.pdf]

Supplementary Material: Screenshots of Patient Educational Tool

The Patient Educational Tool is also accessible via the NCCC Landing Page: https://www.nccc-online.org/understanding-cervical-cancer-screening/as the tool is also equipped with audio-function.

Initial Setup Screen to select into the Normal Pap Smear (Check Up) or Abnormal Pap Smear (Abnormal Pap) Modules

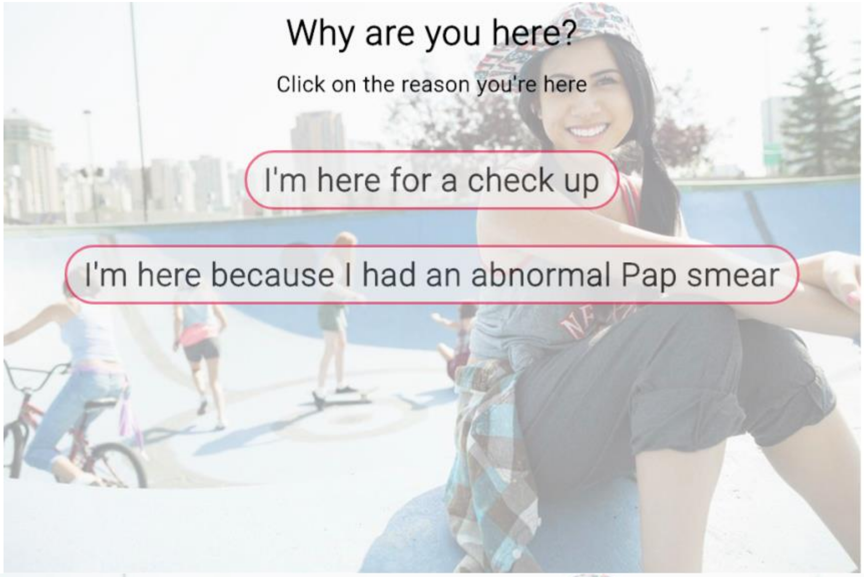

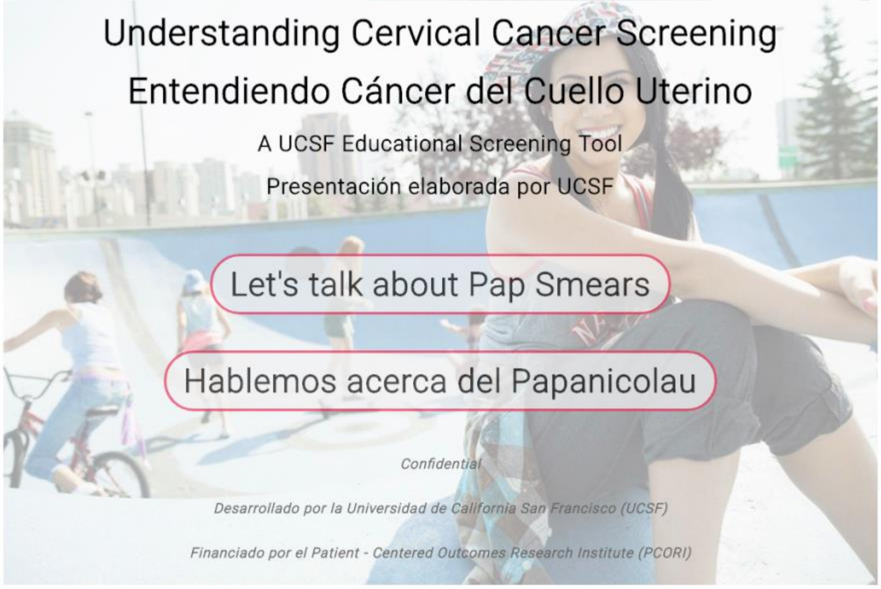

Module: "I'm here for a check up" – English Language

Screenshots of Patient Educational Tool

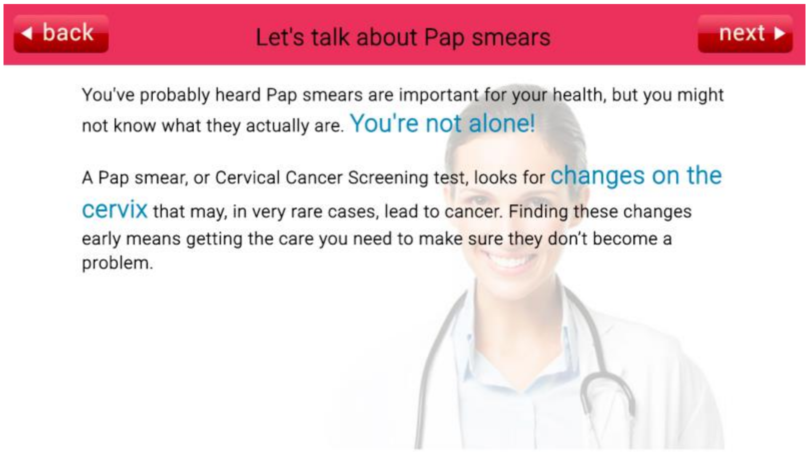

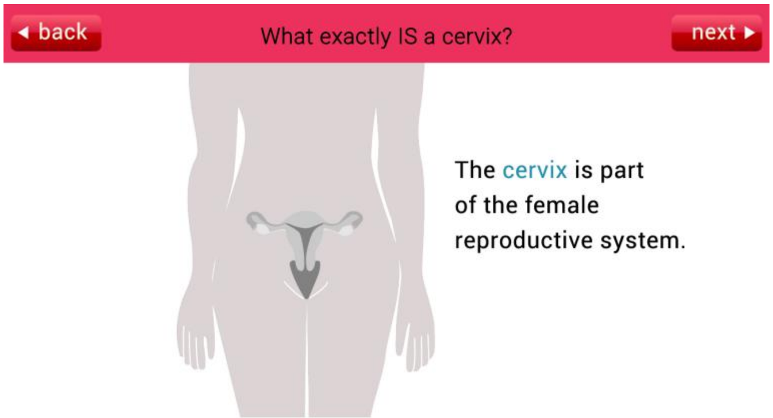

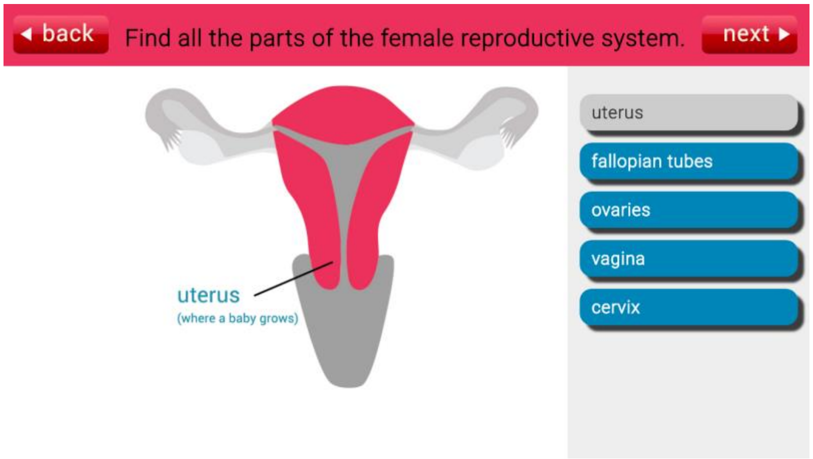

Screenshots of Patient Educational Tool

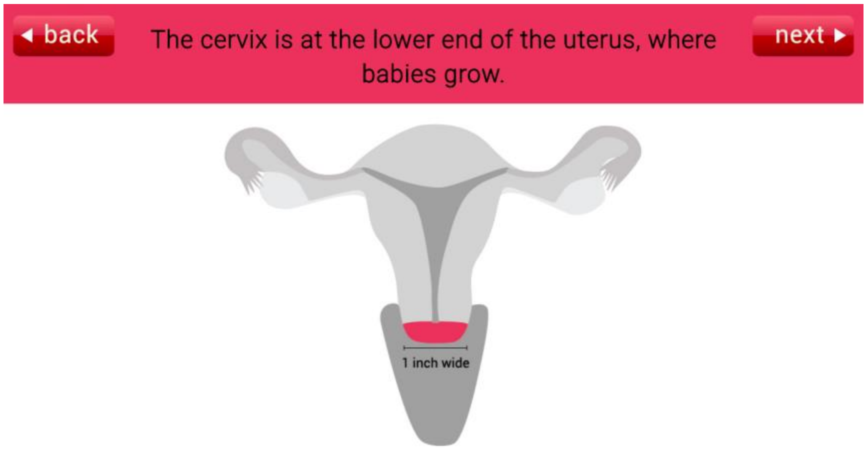

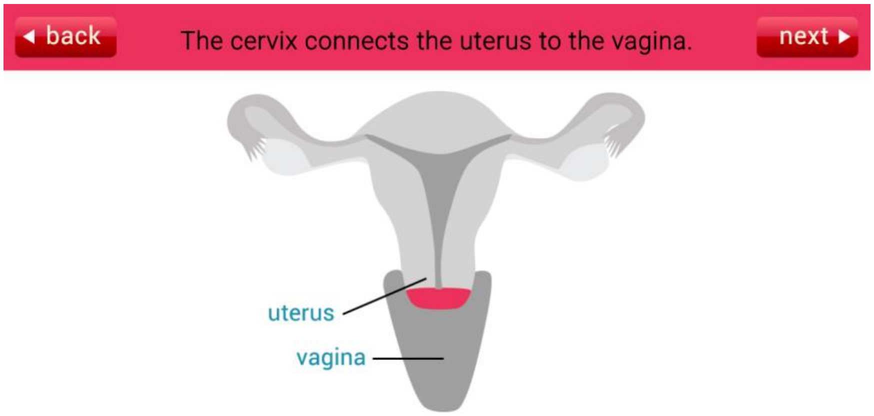

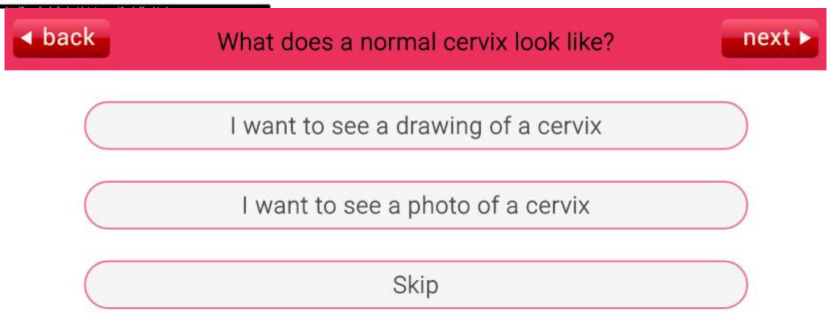

Screenshots of Patient Educational Tool

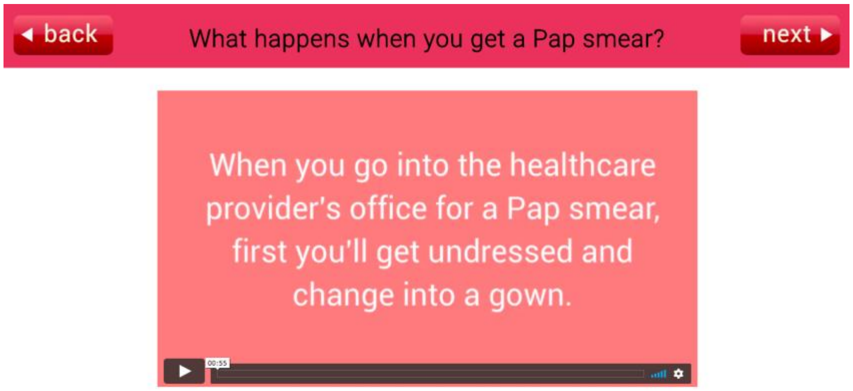

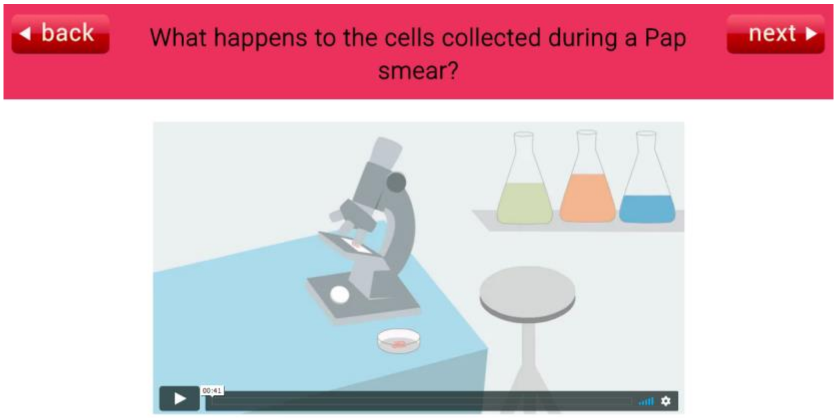

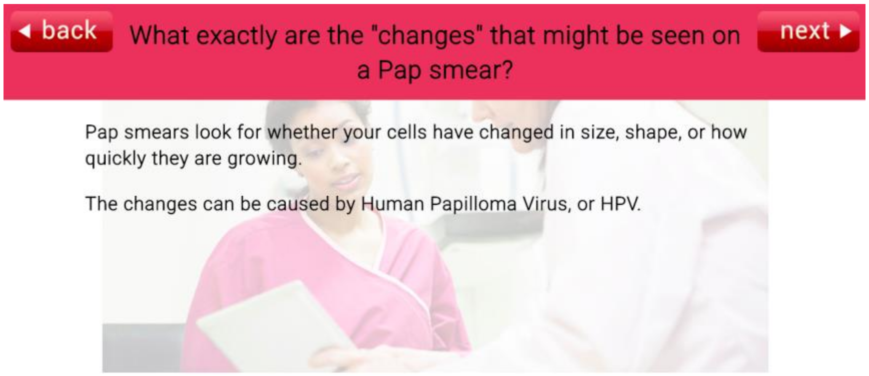

Screenshots of Patient Educational Tool

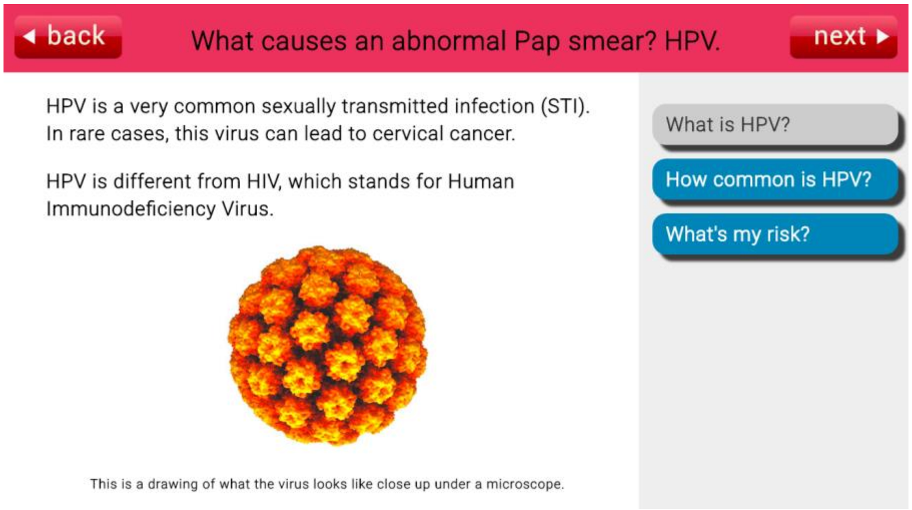

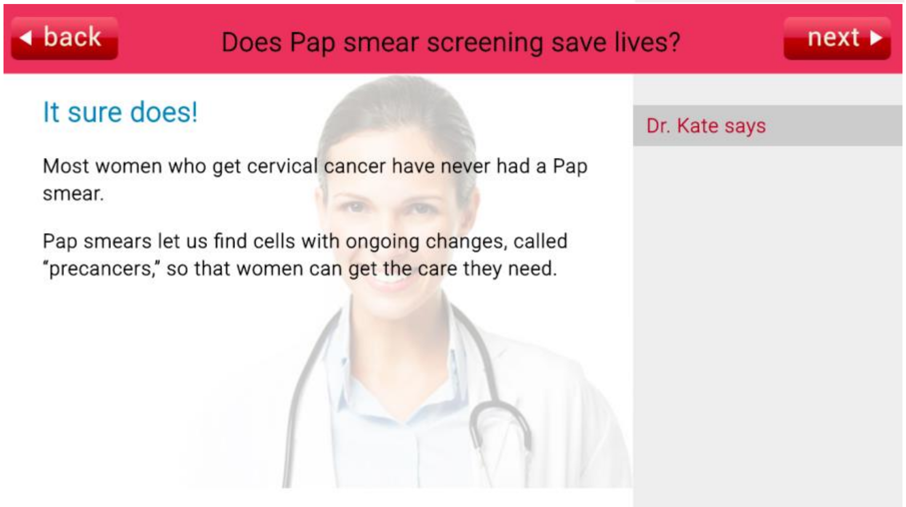

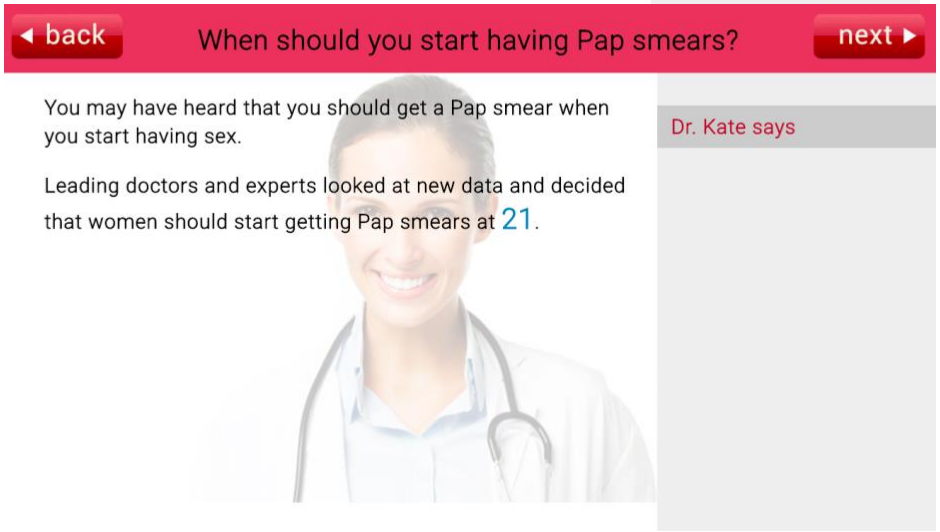

Screenshots of Patient Educational Tool

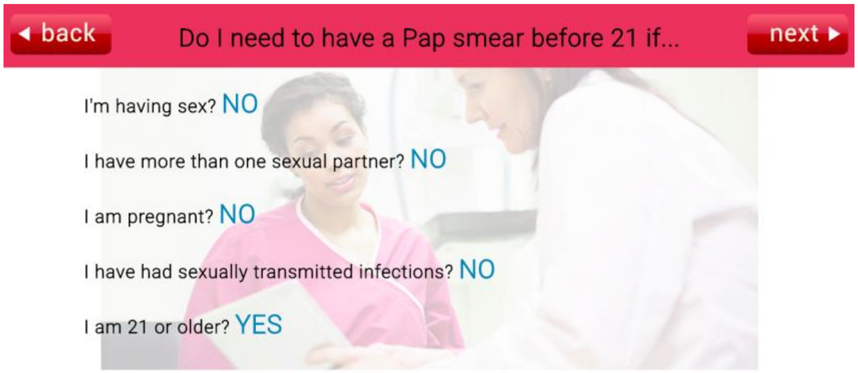

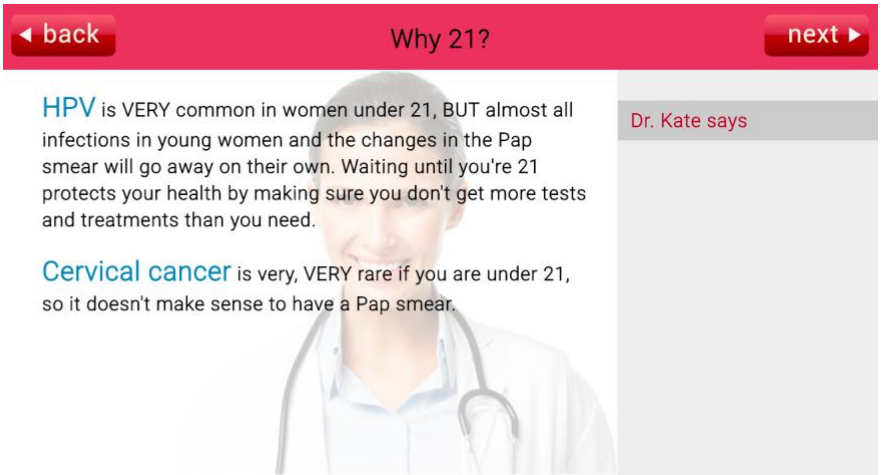

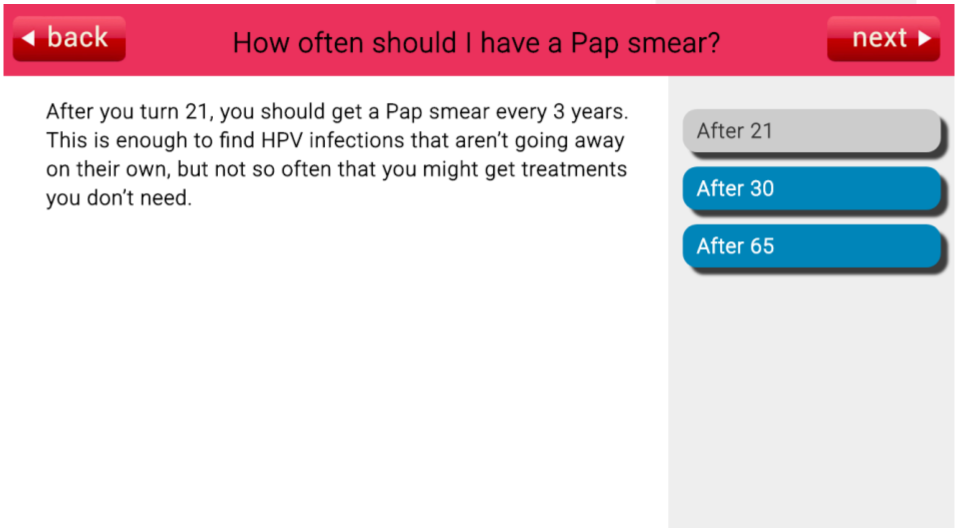

Screenshots of Patient Educational Tool

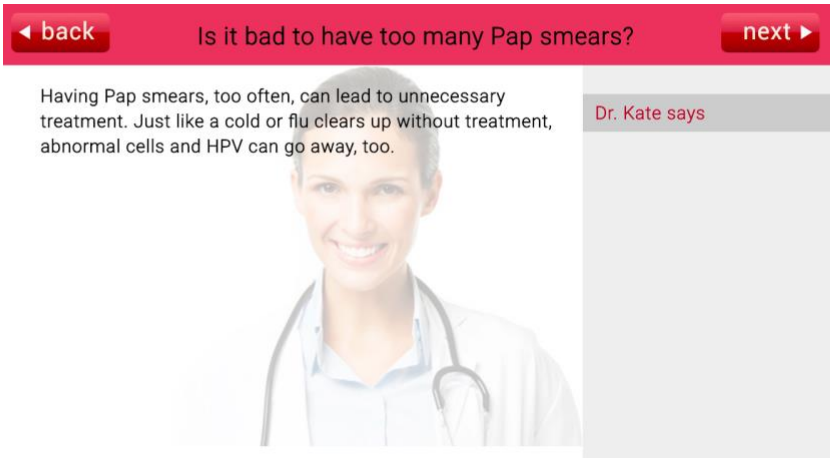

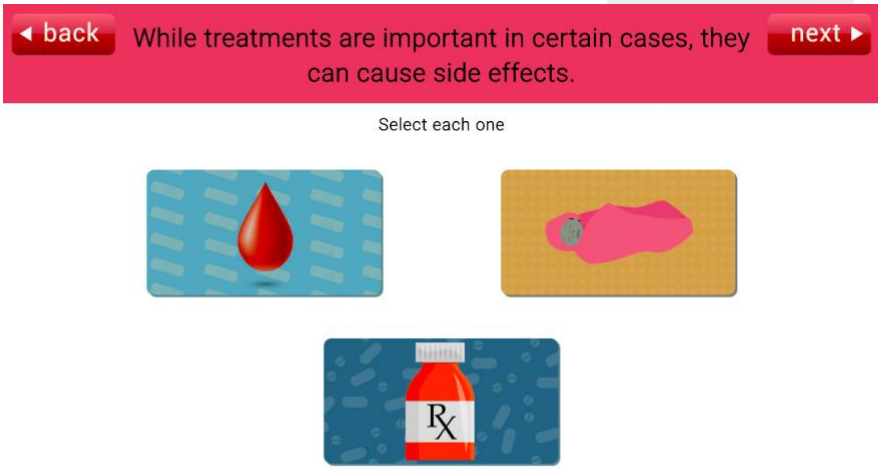

Screenshots of Patient Educational Tool

Module: "I'm here for a check up" – Spanish Language

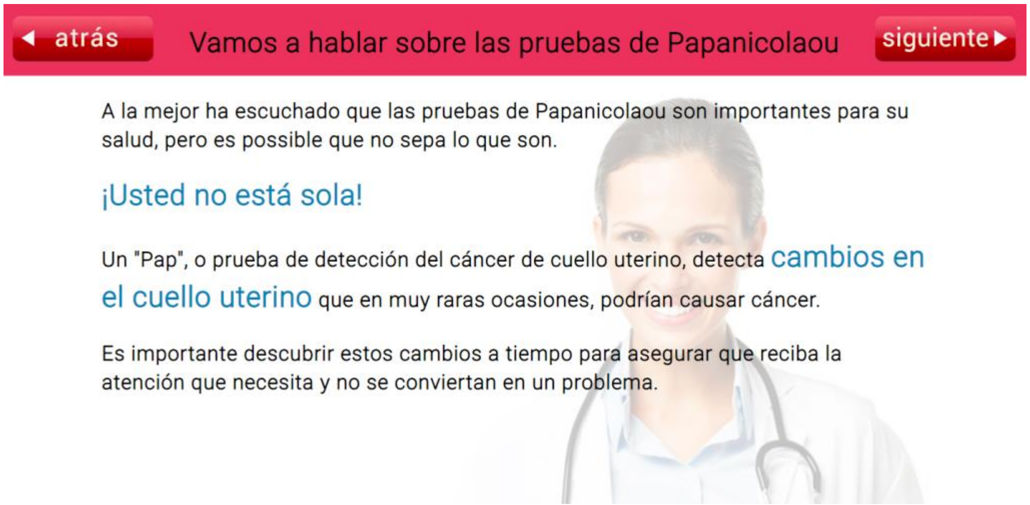

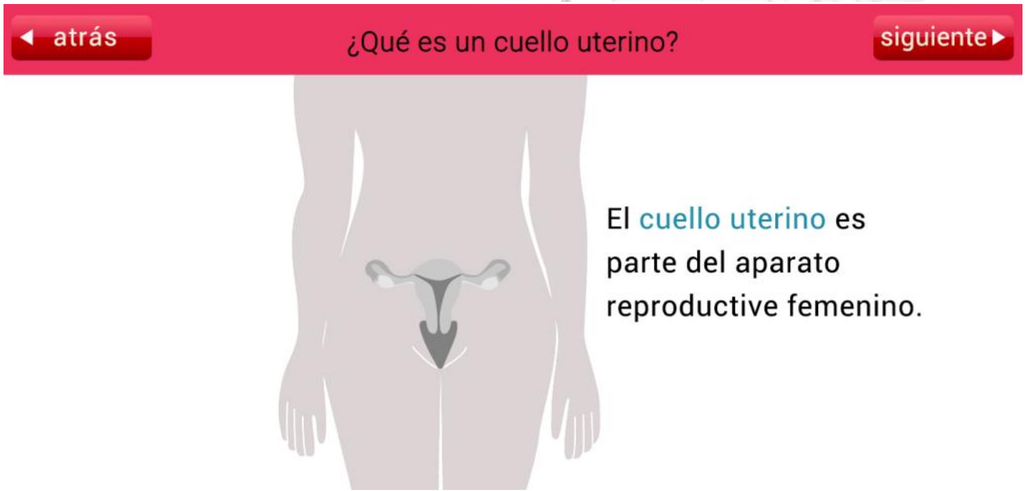

Screenshots of Patient Educational Tool

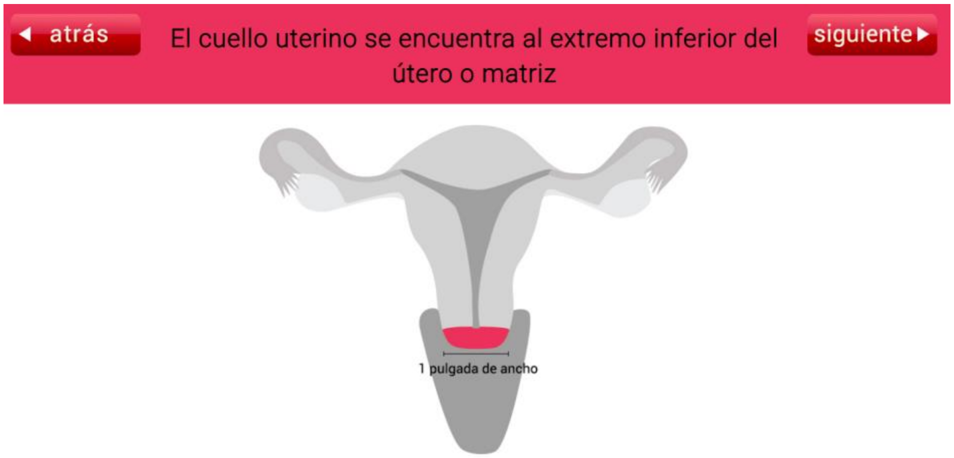

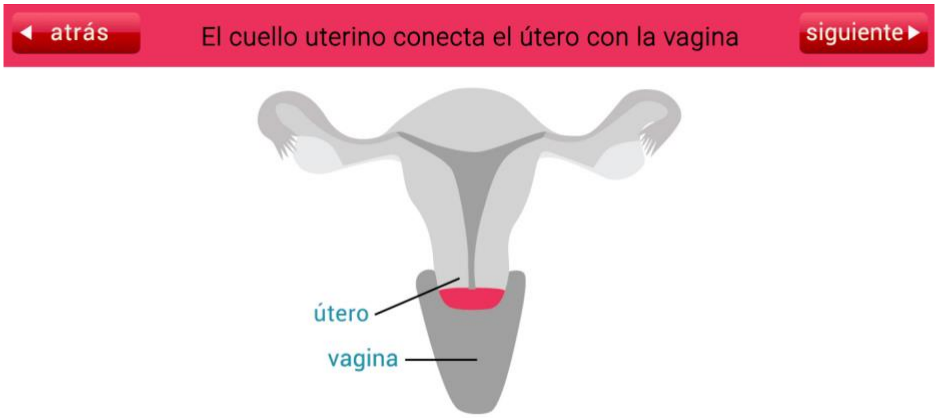

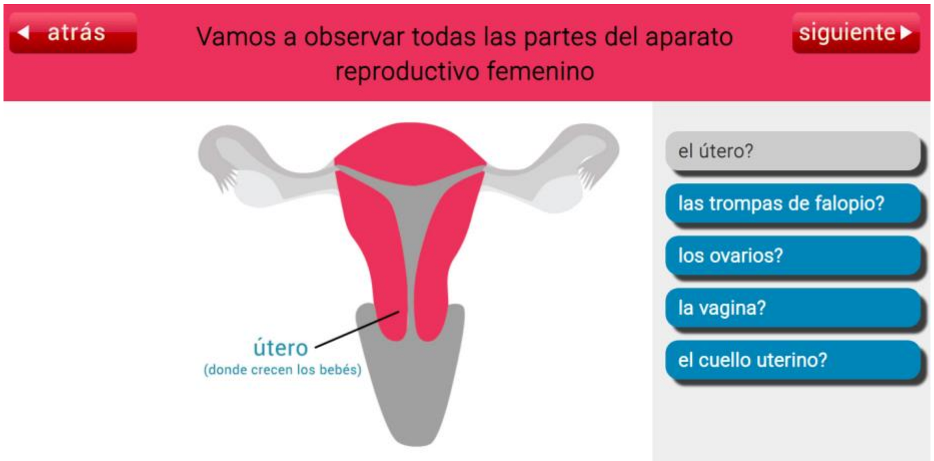

Screenshots of Patient Educational Tool

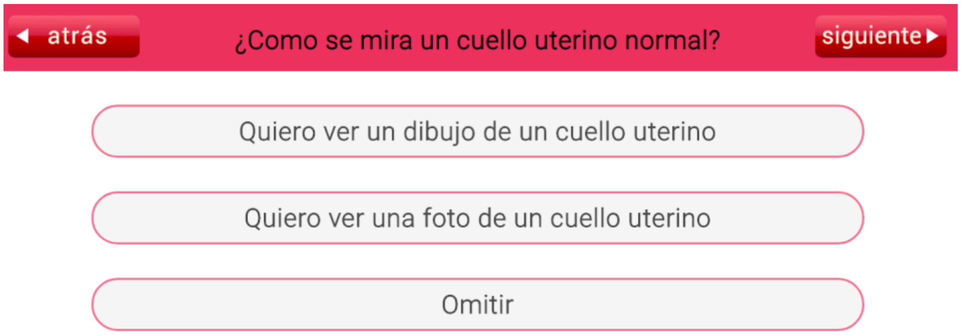

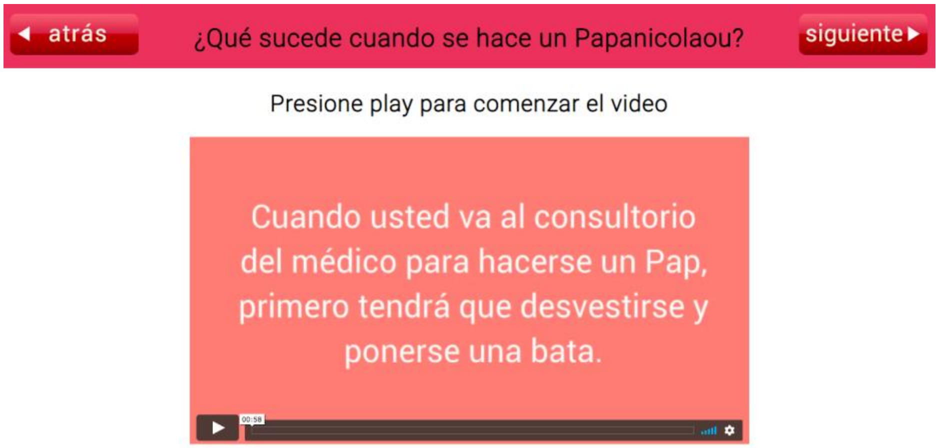

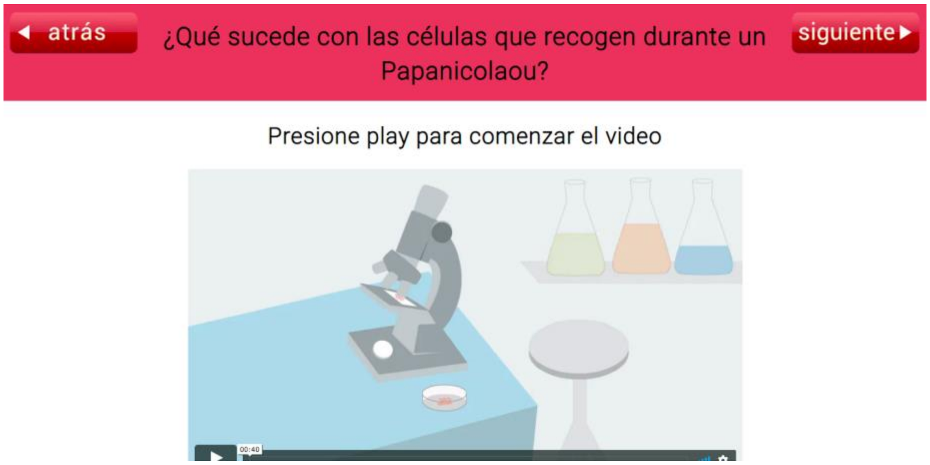

Screenshots of Patient Educational Tool

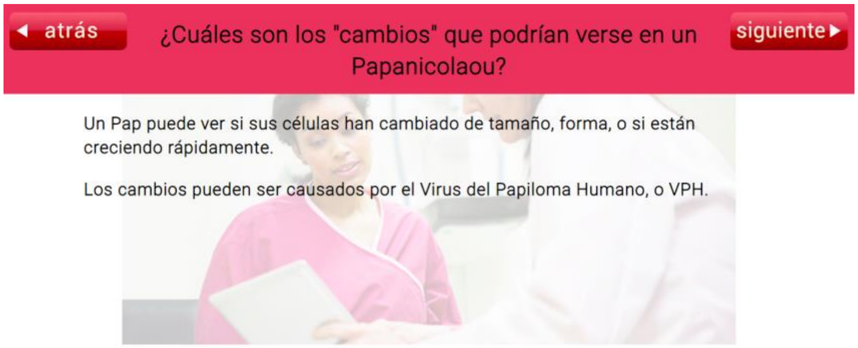

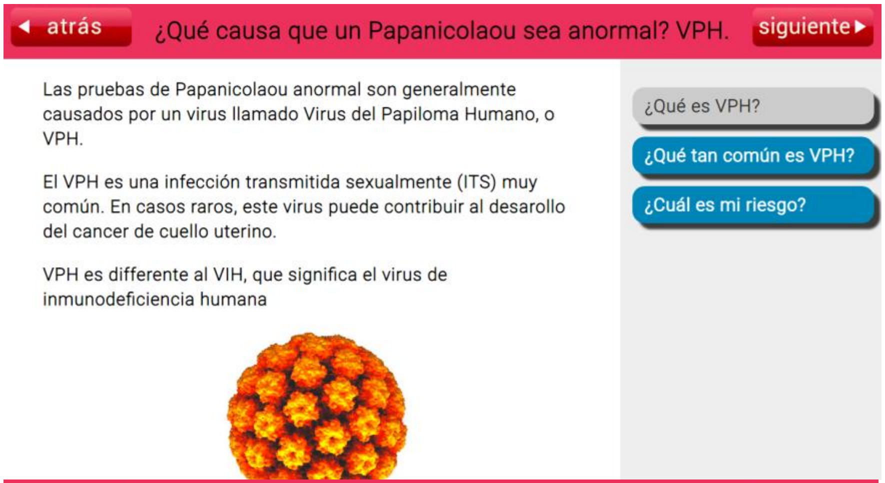

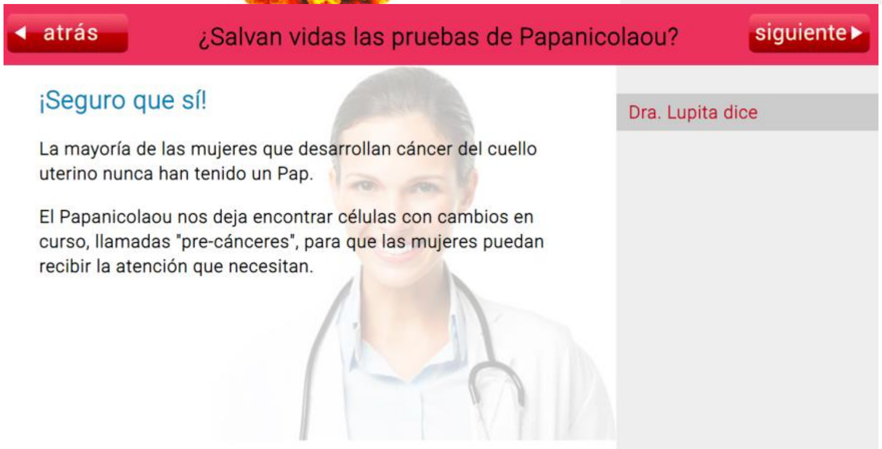

Screenshots of Patient Educational Tool

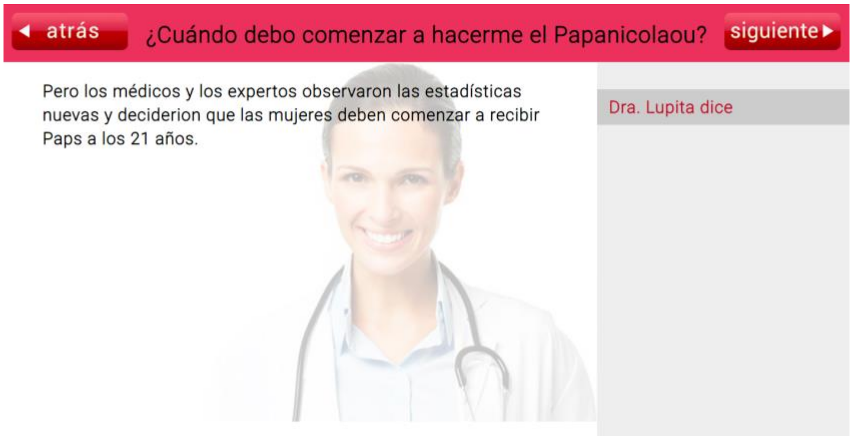

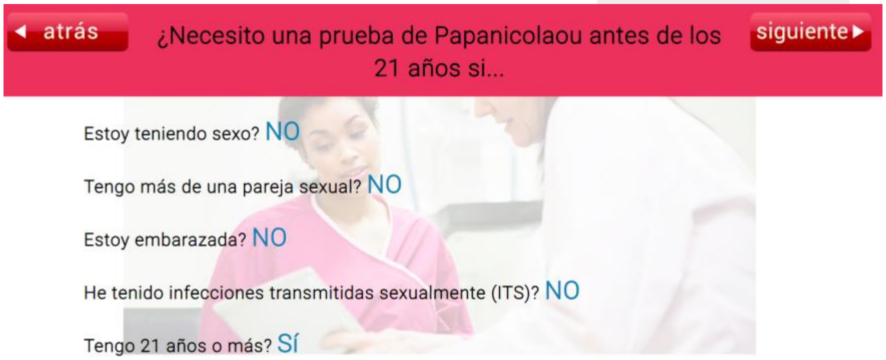

Abnormal Pap Module – English Language

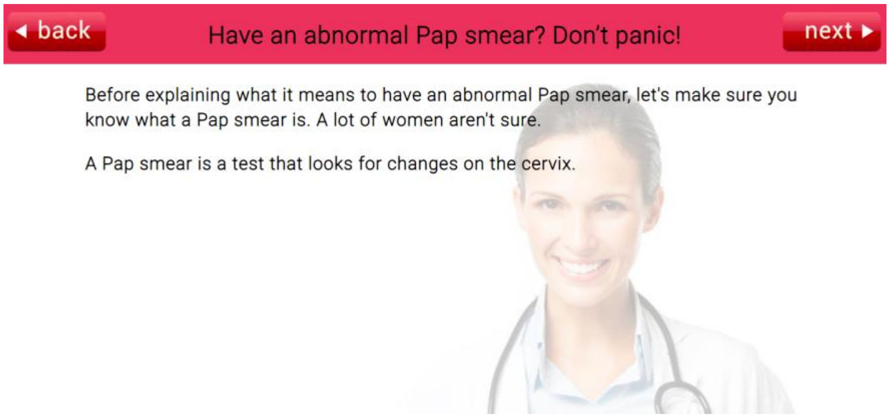

Screenshots of Patient Educational Tool

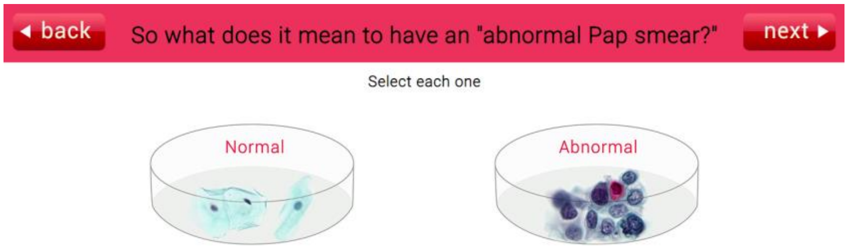

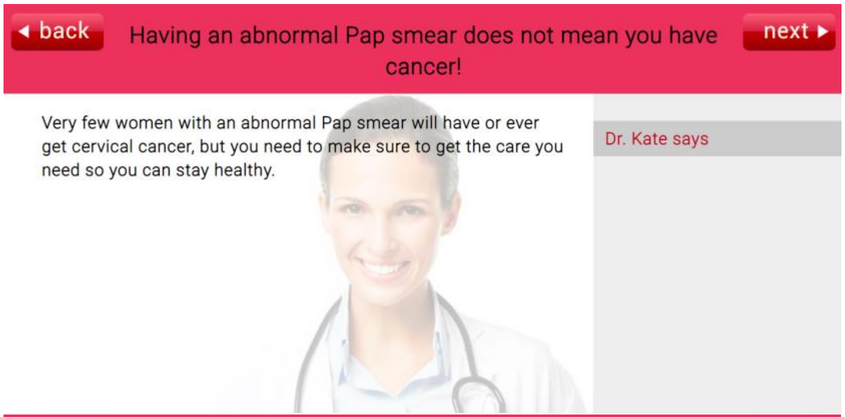

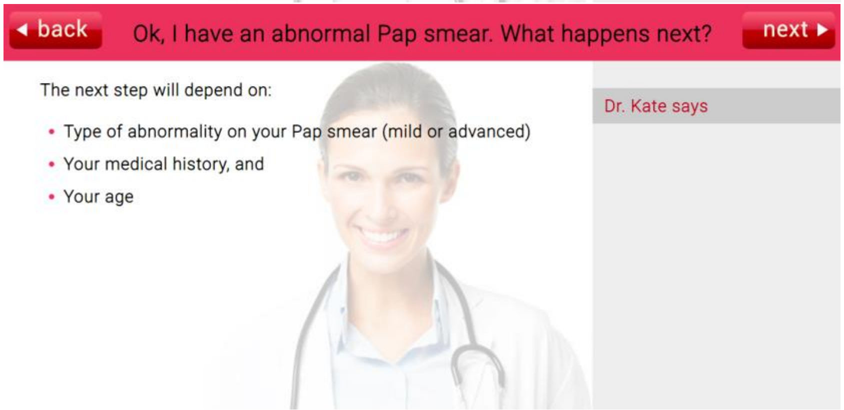

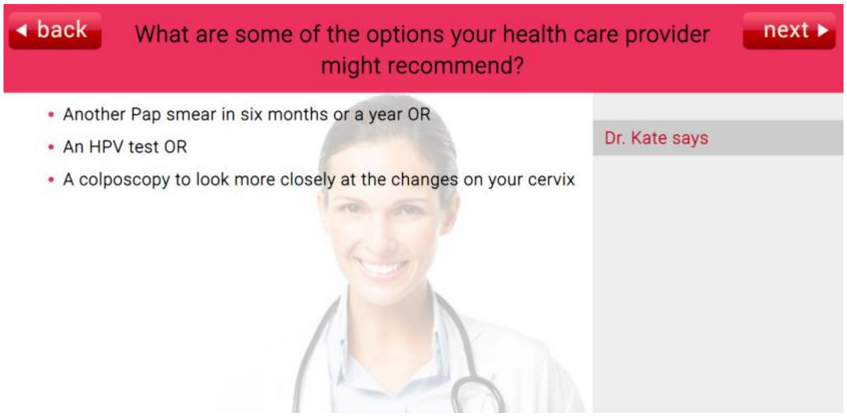

Screenshots of Patient Educational Tool

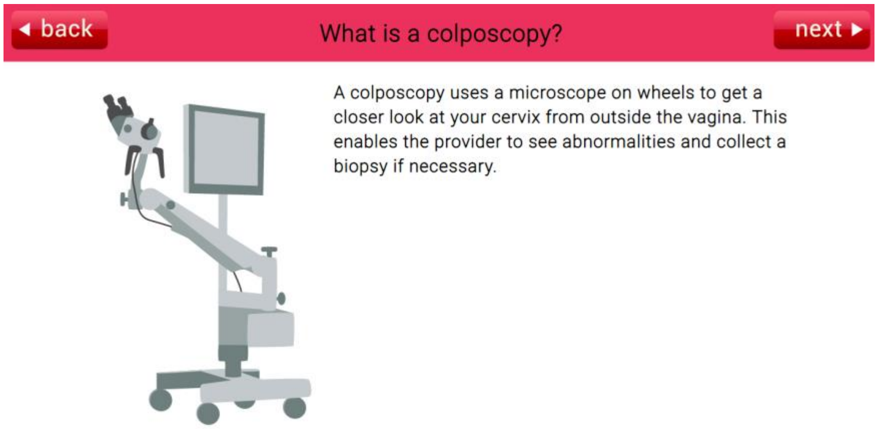

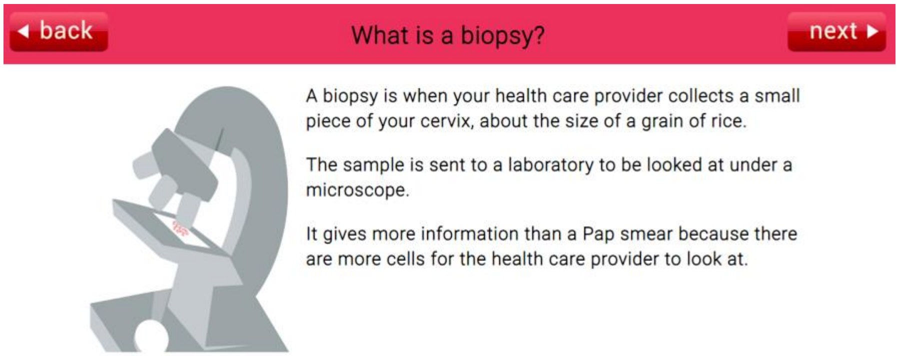

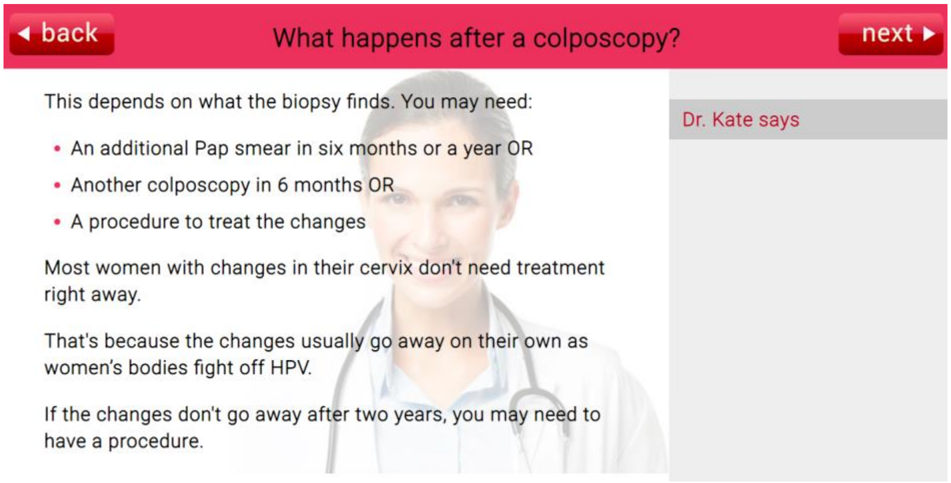

Abnormal Pap Module – Spanish Language

Screenshots of Patient Educational Tool

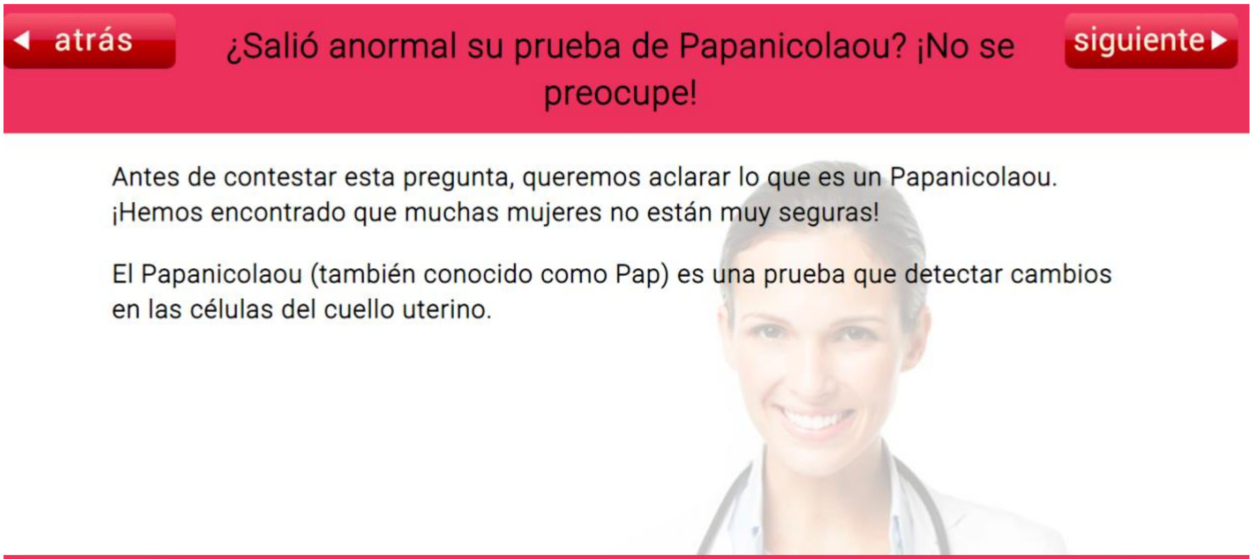

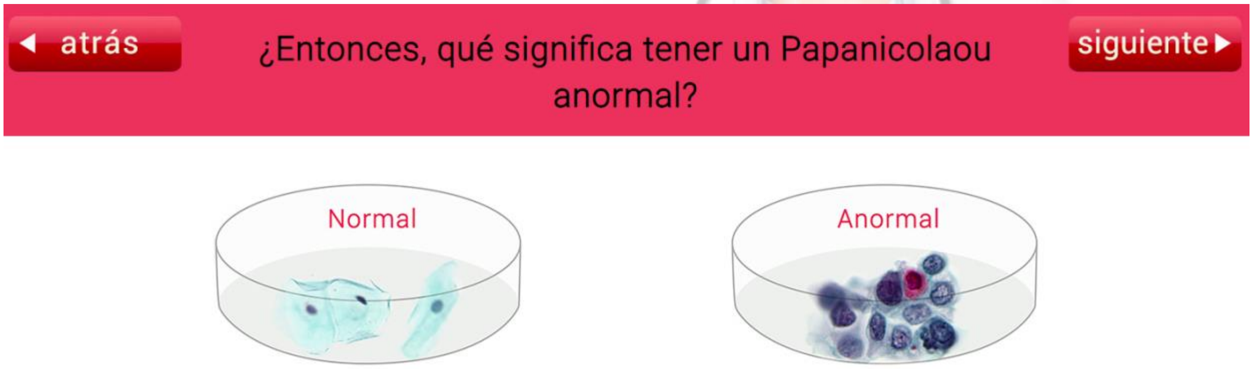

Screenshots of Patient Educational Tool

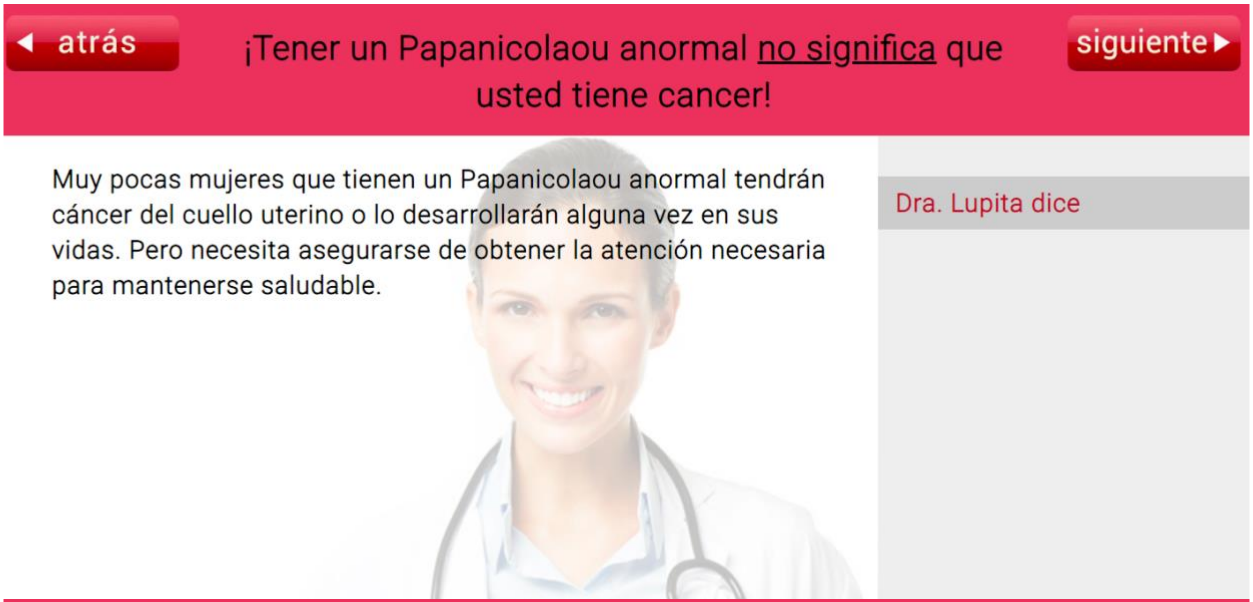

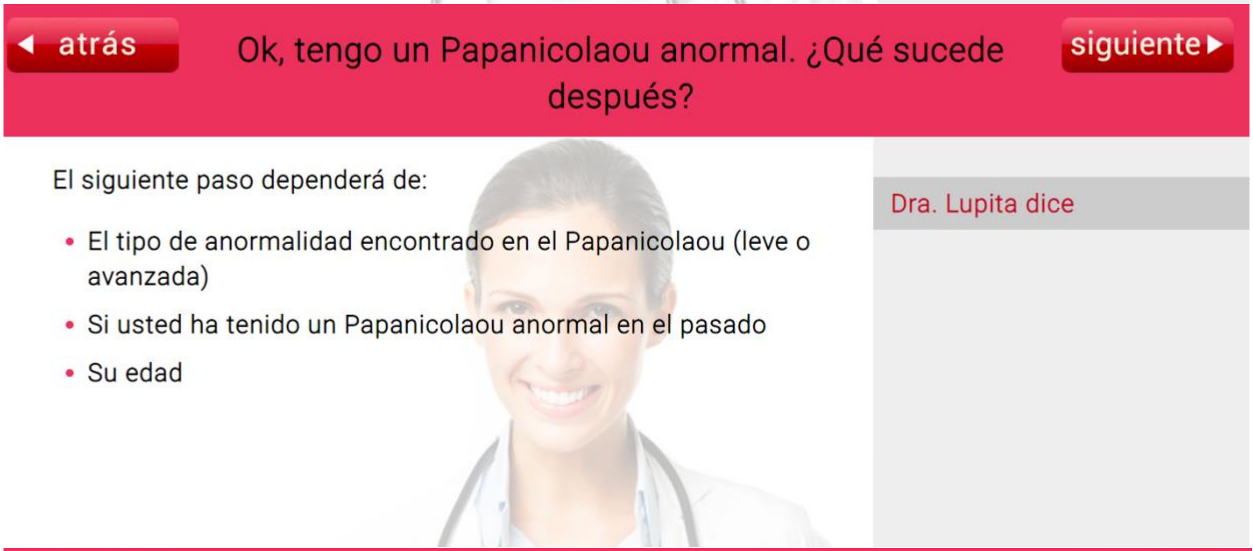

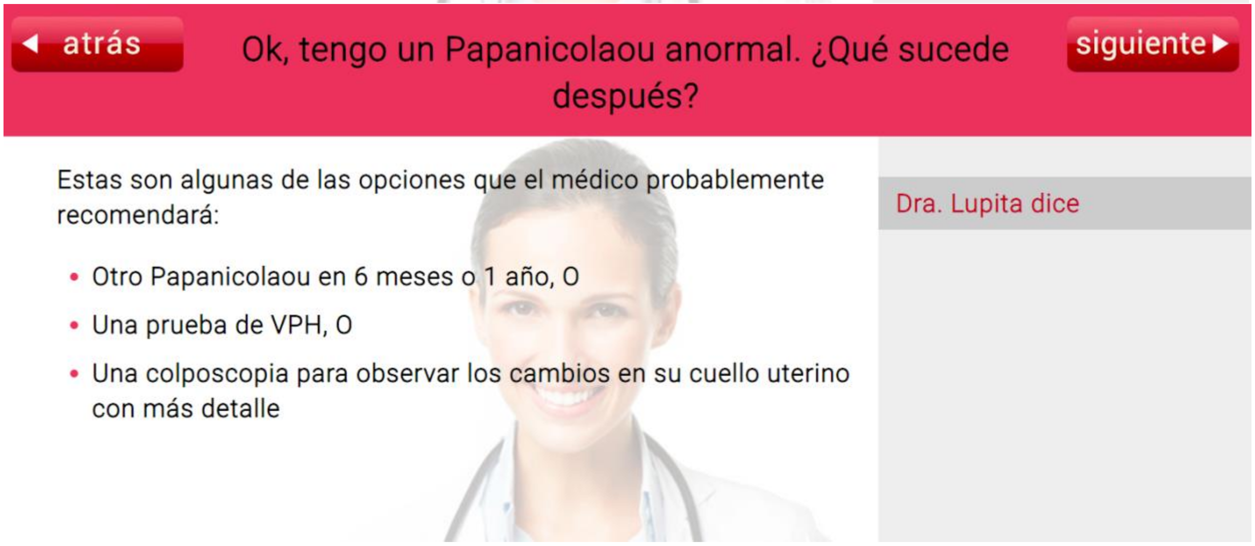

Screenshots of Patient Educational Tool

◄ atrás

¿Qué es una colposcopia?

siguiente ►

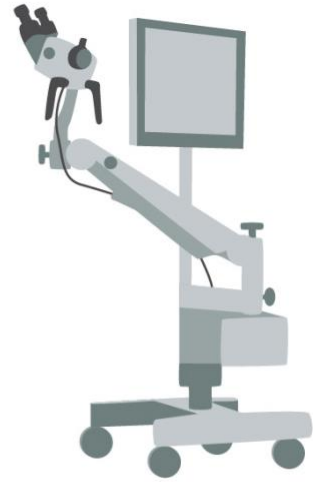

Una colposcopia utiliza un microscopio sobre ruedas para obtener una mirada más cercana a su cuello uterino por fuera de la vagina. Esto permite al médico ver las anormalidades y recoger una biopsia si es necesario.

◄ atrás

¿Qué es una biopsia?

siguiente ►

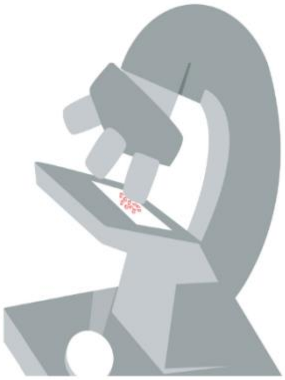

Una biopsia es cuando el médico recoge una muestra pequeña de su cuello uterino, aproximadamente del tamaño de un grano de arroz.

Luego, la muestra se envía a un laboratorio para ser analizada bajo un microscopio.

Esto brinda más información que una prueba de Papanicolaou porque hay más células que su doctor puede mirar.
